# Cannabis-Derived Compounds Cannabichromene and Δ9-Tetrahydrocannabinol Interact and Exhibit Cytotoxic Activity against Urothelial Cell Carcinoma Correlated with Inhibition of Cell Migration and Cytoskeleton Organization

**DOI:** 10.3390/molecules26020465

**Published:** 2021-01-17

**Authors:** Omer Anis, Ajjampura C. Vinayaka, Nurit Shalev, Dvora Namdar, Stalin Nadarajan, Seegehalli M. Anil, Ofer Cohen, Eduard Belausov, Jacob Ramon, Einav Mayzlish Gati, Hinanit Koltai

**Affiliations:** 1 Department of Urology, Sheba Medical Center, 52621 Ramat Gan, Israel; omerwn@gmail.com (O.A.); Jacob.ramon@sheba.health.gov.il (J.R.); 2 Institute of Plant Science, Agriculture Research Organization, Volcani Center, 7505101 Rishon LeZion, Israel; ac.vinayaka@gmail.com (A.C.V.); nuritsh@volcani.agri.gov.il (N.S.); dvoran@volcani.agri.gov.il (D.N.); stalin.rajan84@gmail.com (S.N.); aniganapath@gmail.com (S.M.A.); eddy@volcani.agri.gov.il (E.B.); 3 Israel Gene Bank, Agricultural Research Organization, Volcani Center, 7505101 Rishon LeZion, Israel; oferc@volcani.agri.gov.il (O.C.); einavm@volcani.agri.gov.il (E.M.G.)

**Keywords:** apoptosis, cannabis, cannabinoids, cancer, F-actin, urothelial carcinoma

## Abstract

*Cannabis sativa* contains more than 500 constituents, yet the anticancer properties of the vast majority of cannabis compounds remains unknown. We aimed to identify cannabis compounds and their combinations presenting cytotoxicity against bladder urothelial carcinoma (UC), the most common urinary system cancer. An XTT assay was used to determine cytotoxic activity of *C. sativa* extracts on T24 and HBT-9 cell lines. Extract chemical content was identified by high-performance liquid chromatography (HPLC). Fluorescence-activated cell sorting (FACS) was used to determine apoptosis and cell cycle, using stained F-actin and nuclei. Scratch and transwell assays were used to determine cell migration and invasion, respectively. Gene expression was determined by quantitative Polymerase chain reaction (PCR). The most active decarboxylated extract fraction (F7) of high-cannabidiol (CBD) *C. sativa* was found to contain cannabichromene (CBC) and Δ9-tetrahydrocannabinol (THC). Synergistic interaction was demonstrated between CBC + THC whereas cannabinoid receptor (CB) type 1 and type 2 inverse agonists reduced cytotoxic activity. Treatments with CBC + THC or CBD led to cell cycle arrest and cell apoptosis. CBC + THC or CBD treatments inhibited cell migration and affected F-actin integrity. Identification of active plant ingredients (API) from cannabis that induce apoptosis and affect cell migration in UC cell lines forms a basis for pre-clinical trials for UC treatment.

## 1. Introduction

Urothelial carcinoma (UC) is the most common cancer of the urinary system. The main goals of current treatment are mitigating disease progression and limiting disease recurrence; and thus, the development of efficient new therapies with acceptable side-effect profiles remains essential. Recently, a large retrospective epidemiological study showed that cannabis use is correlated with decreased risk of UC [1].

*Cannabis sativa* has more than 500 constituents, among them more than 150 phytocannabinoids and hundreds of terpenes and flavonoids [2,3,4]. Phytocannabinoids are known to have potential anti-cancer activity [5,6]. Previously, it was shown that phytocannabinoids can prevent proliferation, metastasis, and angiogenesis, as well as induce apoptosis in a variety of cancer cell types including breast, lung, prostate, skin, intestine and glioma [7,8]. This is at least partially due to their ability to regulate signaling pathways critical for cell growth and survival [9,10]. 

In humans, cannabinoid receptor type 1 (CB1) and CB2 were expressed in the urothelium [11]. Importantly, activation of cannabinoid receptor type 2 (CB2) was shown to affect bladder cancer cell viability [12]. CB1 and CB2 receptors were detected in bladder cancer specimens; however, only CB2 was more highly expressed in the tumor than in normal tissue. Bladder cancer cell proliferation was inhibited by treatment with CB2 agonists. This treatment also led in bladder cancer cells to down-regulation of Akt phosphorylation, to caspase 3-activation and to modifications in cellular sphingolipid metabolism. Differences in sphingolipid metabolism were suggested to be linked to cytoskeletal changes and altered cell migration in bladder cancer cells [12]. 

However, little is known regarding various cannabis compounds and/or combinations of these compounds that present anti-cancer activity specifically against UC. Also, in several cases isolated cannabis compounds were found to be less effective than the unrefined content of cannabis inflorescence containing multiple compounds [13,14,15]; therefore, interactions between active cannabis compounds should also be examined. 

In this study, we identified the active plant ingredients (API) from cannabis that have substantial cytotoxic activity against UC cells, determined the API synergistic interaction and part of their mode of action. This research forms a solid basis for future pre-clinical trials for cannabis-based treatments against urinary system cancers.

## 2. Results

### 2.1. Cytotoxic Activity of IGB Cannabis Strain Extracts and Fractions Against Urothelial Cell Carcinoma

**Figure 1:**
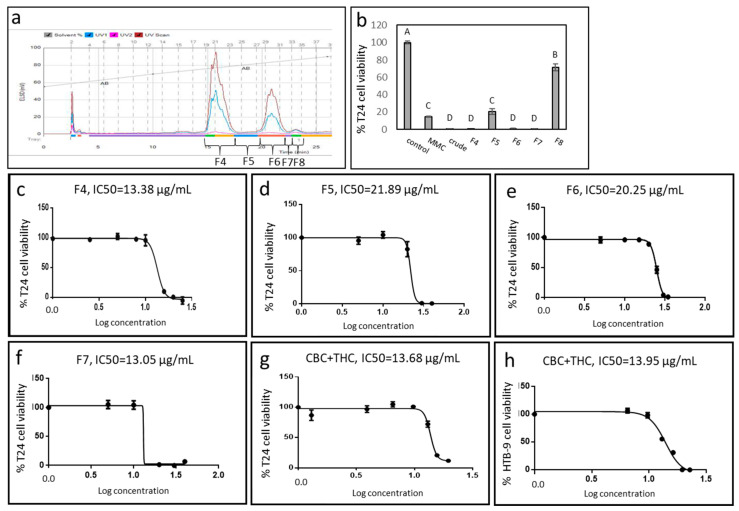
(**a**) Flash chromatography profile of *C. sativa* IGB strain crude extract. Fractions were collected and designated F4-F8. (**b**) Cell viability of T24 cells treated with different fractions of *C. sativa* IGB strain extract and fractions. Cell viability was determined by XTT assay as a function of live cell number. Cells were treated with the crude extract and fractions F4, F5, F6, F7 and F8 at a concentration of 30 μg/mL for 48 h. Mitomycin-C (MMC, 8 µg/mL) served as a positive control. Methanol (control) treatment served as a solvent (vehicle) control. Error bars indicate ± SE (*n* = 3). Levels with different letters are significantly different from all combinations of pairs by Tukey–Kramer honest significant difference (HSD; *p* ≤ 0.05). (**c**–**g**) Dose-effect curves of the crude extract, fractions F4, F5, F6, F7 and CBC + THC on the viability of the T24 cell line. (**h**) Dose-effect curves of crude extract of CBC + THC on the viability of HTB-9 cell line. Data points were connected by non-linear regression lines of the sigmoidal dose-response relation. GraphPad Prism was used to produce the dose-response curve and IC50 doses.

The ethanol extract of fresh inflorescence of a high-CBD cannabis strain from Israel Gene Bank (IGB) was fractionated using Flash chromatography (Figure 1a). The crude extract showed cytotoxic activity against UC cell line T24 (Figure 1b). Some extract fractions (F4, F6, F7) showed significantly increased activity in comparison to the vehicle control, and to 8 µg/mL Mitomycin-C (MMC; Figure 1b). The IC50 of F4, F5, F6 and F7 were 13.38, 21.89, 20.25 and 13.05 µg/mL, respectively (Figure 1c–f). Another fraction (F8) had only minor cytotoxic activity (Figure 1b). F4-F7 were also highly active on another UC cell line, HTB-9 (Supplementary Figure S1). 

### 2.2. Active Fraction Chemical Composition and Determination of Activity

The active fractions (F4–F7) were subjected to chemical analysis (Supplementary Table S1). The most active fraction F7 contained CBC (85.8%) and THC (14.2%). In comparison, the crude extract consisted of 3.0% CBC and 26.4% THC (Supplementary Table S1). GC/MS analysis showed no terpenes in F7. A combination of CBC and THC standards at the ratio found in F7 (~6:1) was active at a level similar to F7 (IC50 of 13.68 and 13.05 µg/mL, respectively; Figure 1f, 1g). The combination CBC+THC (~6:1) showed similar cytotoxic activity on HTB-9 (IC50 of 13.95 µg/mL; Figure 1h).

### 2.3. Synergistic Interactions between CBC and THC 

**Figure 2:**
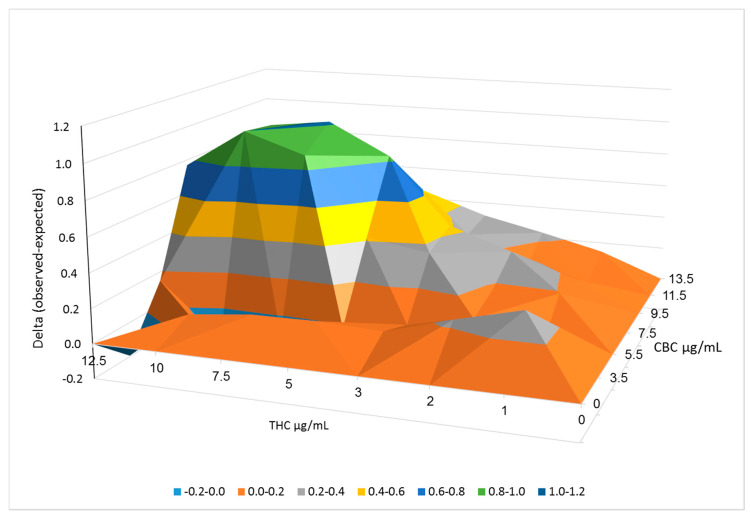
Synergistic interactions between CBC and THC on cell viability of T24 cells following combined treatments. Synergy of cytotoxic activity calculated using the Bliss independence drug interaction model. Synergy is apparent when the experimental (observed) value of cell death is higher than the calculated (expected) value. Delta between the observed and expected values, calculated using the Bliss model are shown in the Y axis.

We determined the synergistic interaction between the two F7 compounds, CBC, and THC (at different ratios and concentrations) on T24 cell viability using the Bliss independence drug interaction model (Figure 2). The outcome of the Bliss model represents the delta between the experimental cell survival (as the fraction of inhibition achieved by the combination of drugs) and the calculated (expected) fraction of inhibition, based on cell survival analysis of CBC or THC alone. The higher delta representing a more profound effect on cell survival achieved by the CBC and THC combinations was apparent mainly at high phytocannabinoid concentrations (Figure 2; Supplementary Table S2).

### 2.4. Determination of CB1, CB2, TRPA1, TRPV1 and TRPV2 Receptor Involvement in Cytotoxic Activity

**Figure 3:**
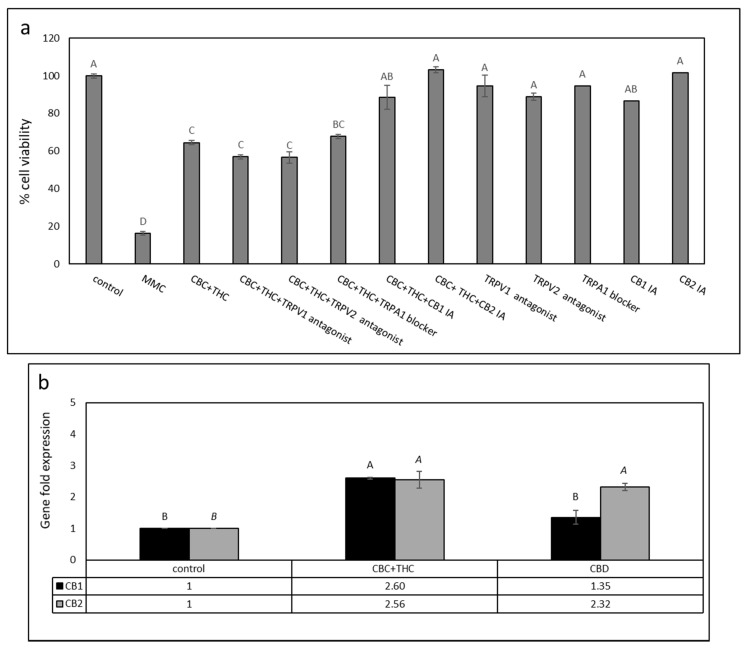
(**a**) Cell viability of T24 cells treated with CBC+THC (11.5 + 2.0 μg/mL, respectively) with or without CB1, CB2 inverse agonists (IA), TRPV1 or TRPV2 antagonists, or a TRPA1 blocker (10 µM). Cell viability was determined by XTT assay. Mitomycin-C (MMC, 8 µg/mL) served as a positive control. Methanol (control) treatment served as a solvent (vehicle) control. Error bars indicate ± SE (*n* = 3). Levels with different letters of the same font are significantly different from all combinations of pairs according to the Tukey–Kramer honest significant difference (HSD). (**b**) Quantitative PCR determination of the RNA steady state level in T24 cell line of CB1 receptor (*CNR1*) or CB2 receptor (*CNR2*) genes, after treatment with CBC + THC (11.2 + 1.8 μg/mL, respectively) or CBD (9.2 μg/mL) for 6 h relative to control. Methanol (control) treatment served as a solvent (vehicle) control. Gene transcript values were determined by quantitative PCR as a ratio between the target gene versus a reference gene (*HPRT1*; geneID 3251). Values were calculated relative to the average expression of target genes in treated versus control using the 2^ΔΔCt^ method.

T24 cells were treated with a mixture of CBC + THC with or without 10 μM of CB1 or CB2 inverse agonists (IA), TRPV1 or TRPV2 antagonists, or a TRPA1 blocker. Treatment with CBC + THC in the presence of CB1 or CB2 IAs (10 μM) significantly decreased cytotoxic activity (Figure 3a). However, treatment of T24 cells with CBC + THC in the presence of the TRPA1 blocker, or TRPV1 and TRPV2 antagonists did not significantly affect cytotoxicity of the phytocannabinoid treatment (Figure 3a). Treatment with the CB IAs, TRPV antagonists or TRPA1 blocker only did not significantly alter cell viability (Figure 3a). 

CBD is known for its anticancer activity [16] and is the abundant phytocannabinoid in the IGB strain (50.7%, Supplementary Table S1). Hence, we also examined the effect of CB1 and CB2 IA on CBD activity. For the CBD treatments, only the addition of CB2 IA completely abolished cytotoxicity (Supplementary Figure S2).

We also examined the expression of CB1 and CB2 receptor genes (i.e., *CNR1* and *CNR2*, respectively), putatively involved with CBC + THC activity, in T24 cells. Both receptor genes are expressed in T24 cells, as determined using *HPRT1* as a reference gene. CB2 receptor gene expression was induced 2.56-fold in the CBC + THC treatment (Figure 3b). Similarly, CB1 receptor gene expression was induced in this treatment 2.6-fold (Figure 3b). Expression of both CB1 and CB2 receptor genes increased by 1.35 and 2.32-fold, respectively, with CBD treatment (Figure 3b). Similar CB1 and CB2 receptor gene expressions with phytocannabinoid treatments were obtained using actin as the reference gene (not shown).

### 2.5. Determination of the Effect of CBC + THC or CBD Treatments on Cell Cycle Arrest and Apoptosis 

**Figure 4:**
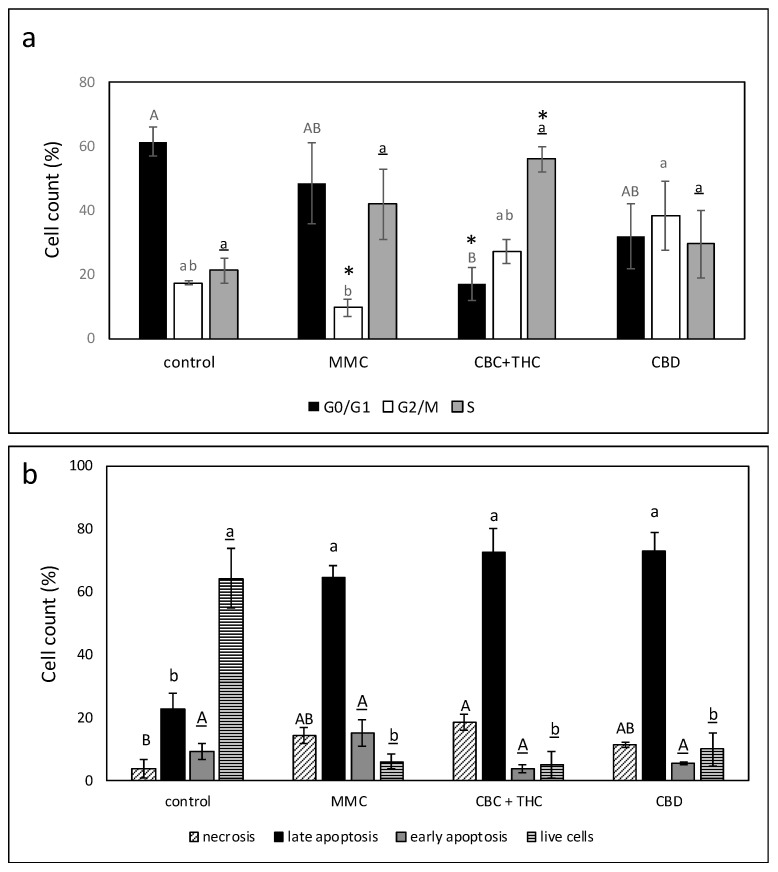
(**a**) Determination of the stages of cell cycle arrest following treatment with CBC + THC (17.2 + 2.8 μg/mL, respectively) or CBD (15 μg/mL) on the T24 cell line for 24 h. Mitomycin-C (MMC, 8 µg/mL) served as a positive control. Methanol (control) treatment served as a solvent (vehicle) control. Error bars indicate ± SE (*n* = 3). Levels with different letters of the same font are significantly different from all combinations of pairs according to Tukey–Kramer honest significant difference (HSD; *p* ≤ 0.05). * indicates significantly different mean from the control based on Student *t*-test (*p* ≤ 0.05). (**b**) Proportion of viable, apoptotic, or necrotic cells following treatment with CBC+THC (17.2 + 2.8 μg/mL, respectively) or CBD (15 μg/mL) on T24 cell line for 48 h. Mitomycin-C (MMC, 8 µg/mL) served as a positive control. Methanol (control) treatment served as solvent (vehicle) control. Error bars indicate ± SE (*n* = 3 for treatments, *n* = 4 for the control). Levels with different letters are significantly different from all combinations of pairs according to Tukey-Kramer honest significant difference (HSD; *p* ≤ 0.05).

CBC + THC treatment led to a significant reduction in G0/G1-phase cells and to a significant increase in the percentage of cells in the S-phase of the cell cycle (56.0%, Figure 4a, Supplementary Figure S3). This is in comparison to 21.2% S-phase cells in the control (Figure 4a). CBD treatment led to some (insignificant) increase in G2/M-phase cells (38.3%) in comparison to 17.4% in the control (Figure 4a). 

CBC + THC treatment of T24 cells for 48 h led to 76.2% cell apoptosis in comparison to 32.0% in the control and 79.6% in MMC-treated cells (Figure 4b; Supplementary Figure S3). All examined treatments led to a minor increase in necrosis in comparison to the control (3.9%), with CBC+THC treatment resulting in 18.5% necrotic cells (Figure 4b; Supplementary Figure S3). An increase in apoptosis with the CBC + THC treatment was already evident at 24 h (Supplementary Figure S4).

### 2.6. Determination of the Effect of CBC + THC and CBD Treatments on Cell Motility 

**Figure 5:**
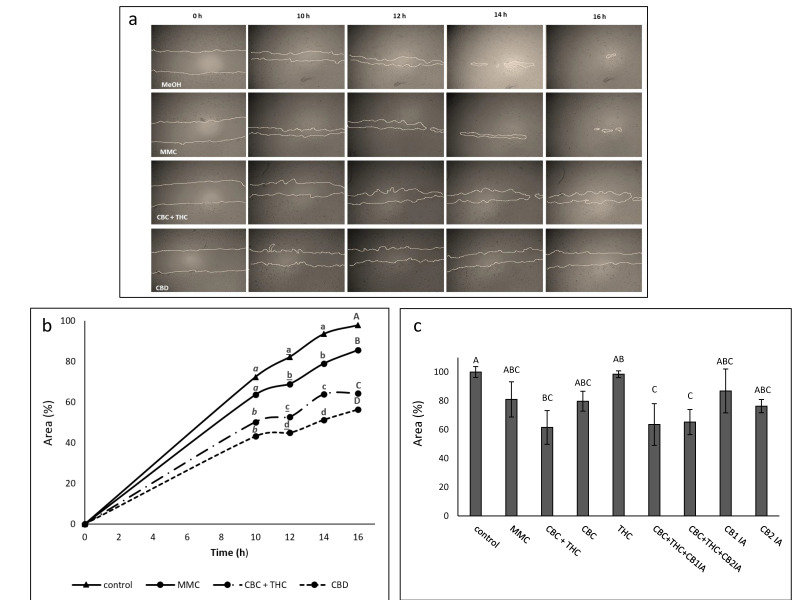
(**a**) Examples of pictures taken for estimation of the treatment effect of CBC + THC (8.6 + 1.4 µg/mL respectively), CBD (10 µg/mL) or Mitomycin-C (MMC, 4 µg/mL) on recovered area of confluent monolayers of T24 cell line at 0, 10, 12, 14 and 16 h. Methanol (control) treatment served as solvent (vehicle) control. (**b**) The effect of treatment with CBC + THC (8.6 + 1.4 µg/mL respectively) or CBD (10 µg/mL) on the recovered area of confluent monolayers of the T24 cell line. (**c**) The effect of treatment with CBC+THC (8.6 + 1.4 µg/mL respectively), CBC (10 μg/mL), THC (10 µg/mL) or CBC+THC (8.6 + 1.4 µg/mL respectively) with CB1 or CB2 IA (10 µM) on the recovered area of confluent monolayers of the T24 cell line at 14 h. Mitomycin-C (MMC, 4 µg/mL) served as a positive control. Methanol (control) treatment served as solvent (vehicle) control. Values are means ± SE (*n* = 12). Values with different letters of the same font are significantly different from all combinations of pairs according to Tukey–Kramer honest significant difference (HSD; *p* ≤ 0.05).

The effects of different compound combinations on cell migration was examined using scratch assays with treatments at sub-lethal concentrations of CBD (10 μg/mL), CBC + THC (8.6 + 1.4 μg/mL, respectively) or MMC (4 μg/mL) (Figure 5a). A marked and significant reduction in the ability of the cells to migrate into the scratch was noted for cells treated with CBD or CBC + THC in comparison to the control (Figure 5b). MMC treatment was effective in reducing cell migration in comparison to the control, but to a lesser extent than CBD or CBC + THC treatments (Figure 5b). CBC at a concentration of 10 μg/mL inhibited cell motility activity, which was reduced (but not significantly) in comparison to CBC + THC. In comparison, THC 10 μg/mL did not inhibit cell migration (Figure 5c). Neither CB1 nor CB2 IAs affected the CBC + THC reduction of cell motility (Figure 5c). 

### 2.7. Determination of the Effect of CBC + THC and CBD Treatments on Cell Invasion 

The effects of the cannabinoid compounds on cell invasion were examined using a transwell assay. At sub-lethal concentrations, CBC + THC treatment reduced cell invasion only to a minor extent (90.5%) relative to vehicle control. Similarly, CBD treatment reduced cell invasion 89.7% in comparison to the control (Table 1). As a positive control, MMC treatment reduced cell invasion by 43.7% in comparison to the vehicle control (Table 1). 

### 2.8. Determination of the Effect of CBC + THC and CBD Treatments on F-actin Filaments 

**Figure 6:**
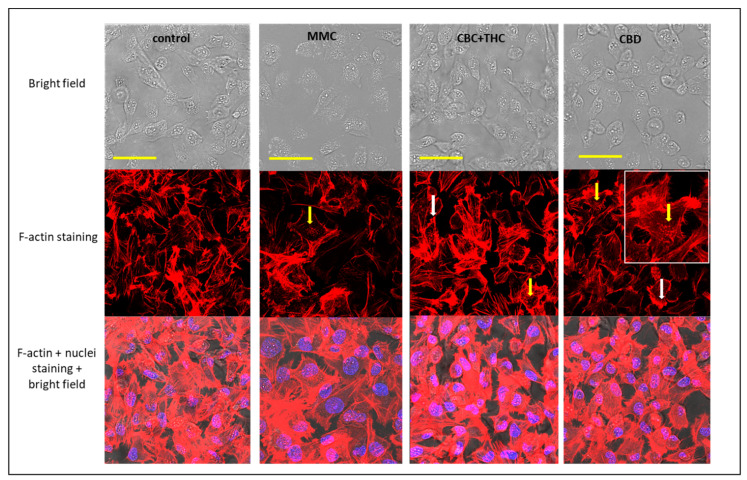
Confocal images of T24 cells following treatment with methanol (the vehicle control), Mitomycin-C (MMC, 4 µg/mL) as positive control, CBC + THC (8.6 + 1.4 µg/mL, respectively) and CBD (10 µg/mL) on T24 cell line for 24 h. F-actin (EasyProbes™ ActinRed 555 Stain, red stain), and nuclei (Hoechst, blue stain) were stained. Bars = 50 µm; yellow arrows point to disintegration of F-actin filaments visualized as characteristic spots; white arrows point to induced accumulation of F-actin filaments on the cell periphery.

Treatment of cells with sub-lethal concentrations of CBC + THC led to disintegration of F-actin filaments visible as characteristic spots (Figure 6, yellow arrows; [17]) and partial disappearance of F-actin filaments inside the cells (Figure 6). Similar effects on F-actin organization were detected for CBD and MMC treatments (Figure 6, yellow arrows), whereas actin filaments remained intact in the vehicle control (Figure 6). In addition, CBC + THC and CBD treatments also induced accumulation of F-actin filaments on the cell periphery (Figure 6, white arrows). 

## 3. Discussion

We identified active plant ingredients (API) from the cannabis extract of a high-CBD strain with significant apoptotic activity against the T24 and HBT-9 UC cell lines. The crude extract, although found to be active, cannot be standardized so that it might be used effectively for clinical treatment. Rather, APIs should be identified for clinical use [15]. To identify the APIs in this study, the crude extract was fractionated and active fractions identified. The most active fraction, F7, contains CBC and THC at approximately a 6:1 ratio. In multiple studies, CBD and THC were previously suggested to possess anticancer activity for a variety of malignancies [18,19], whereas CBC was mostly associated with anticancer activity on prostate carcinomas [20]. 

In the crude IGB strain extract, CBD was abundant in comparison to CBC. Indeed, CBC concentrations in most cannabis strains rarely exceed 0.2–0.3% on a dry weight basis, much lower than the other “major” phytocannabinoids [21]. Yet, CBC + THC (the latter in minute amounts) acts similarly to CBD in terms of cytotoxicity (i.e., comparable concentrations are needed for significant cytotoxicity). Thus, CBC possesses an interesting biological activity with potential for medical use. Additionally, these results demonstrate the ability of the methodological approach taken here—i.e., fractionation and determination of active fractions (e.g., F5 or F7)—to identify highly active molecules and/or combinations thereof present in minute amounts, especially those which have their activity masked by the more abundant phytomolecules (CBD in this case). The methodological approach taken here may also identify more than one combination; for example, F5 and F6 show similar IC50 but have different compositions. 

In this study, synergistic interaction between CBC and THC was established. The synergistic interaction between these two cannabis compounds was dependent on specific ratios and might be related to the entourage effect, described previously as the superior medical activity of phytomolecules in cannabis chemovars vs. single molecules [13,14,15]. 

CB2 IA significantly and completely blocked CBC + THC and CBD cytotoxicity, suggesting that the CB2 receptor may be associated with this activity. CB1 receptor IA reduced only CBC + THC activity, suggesting that the CB1 receptor may be involved to a minor extent in CBC+THC activity on T24 cells. We found that CB1 and CB2 receptor genes are expressed in T24 cells; gene expression of CB1 and CB2 was previously found in various studies to be correlated with the presence and function of receptor proteins [12,22,23]. Accordingly, the CB2 receptor was highly expressed in bladder cancer cells and bladder tumors [12]. Moreover, THC was previously shown to bind and activate CB receptors [24]. CBD was found in earlier studies to be a non-competitive negative allosteric modulator of CB1 [25] and to act as a CB2 IA [26]. In agreement with our findings, the involvement of the CB2 receptor in urothelial malignancy was previously established [12]. 

Interestingly, CB1 and CB2 receptor expression was significantly induced in our study upon treatment with CBC + THC or CBD. The overexpression of cannabinoid receptors CB1 and CB2 correlates with improved prognosis in hepatocellular carcinoma [27]. Following the same line, the up-regulation of CB1 and CB2 receptors by the CBC + THC or CBD treatments in our study may suggest some form of a “positive feedback loop” in which the treatments enhance anti-cancer activity by inducing the expression of cannabinoid receptors, at least in vitro. However, demonstrating the effect of CBC + THC on CB2 and/or CB1 receptors in urothelial carcinoma cells necessitates additional studies at the protein and functional (gain-of-function and/or loss-of-function) levels. 

It was previously suggested that TRPA1 is involved with various pathological conditions of the bladder [28] and that it interacts with CBC as an agonist [26]. In UC, it has been demonstrated that the expression and activity of both TRPV1 and TRPV2 affect tumor stage progression and cell differentiation [29,30]. Expression of TRPV1 decreases progressively as tumor stage increases, and receptor expression possibly correlates to cell differentiation [29]. TRPV2 activation induced apoptosis in low differentiated (high grade) T24 cells [30]. Also, both TRPV1 and TRPV2 interact with phytocannabinoids, including THC and CBC (to a lesser extent) [31]. Nevertheless, in this study neither the TRPA1 blocker, nor the TRPV1 and TRPV2 antagonists reduced CBC + THC or CBD activity, suggesting TRPA1, TRPV1 and TRPV2 are not involved in the cytotoxic activity of these compounds on T24 cells.

Treatment with the synergistic combination of CBC + THC or with CBD led to an increase in apoptotic cell death in T24 cells. These results are in line with other studies showing cannabinoids often induce apoptosis in cancer cells and inhibit cancer cell proliferation [19]. CB2 activation led to ceramide-mediated bladder cancer cell apoptosis [9,10] and treatment of bladder cancer cells with CB2 agonists induced caspase 3-activation [12], adding credence to the suggestion that cannabinoids act, directly or indirectly, via the CB2 receptor in these cells to induce programmed cell death. 

CBC + THC treatment was also associated with S-phase arrest in T24 cell cycle, leading in many cases to apoptosis [32]. In other studies, MMC treatment led to S-phase arrest [33], although this was not significantly observed in our study. CBD treatment on the other hand, unlike CBC + THC, did not lead to S-phase enrichment in T24 cells, suggesting that CBC + THC and CBD affect cells somewhat differently. 

CBC + THC and CBD significantly reduced cell motility in scratch assays. CBC was less active in comparison to the CBC + THC mixture (at equal concentrations) whereas THC at the equivalent mixture concentration showed no inhibition of cell motility. Although activation of CB2 receptors was previously shown to reduce cell motility in bladder cancer cells [12], in our study neither CB2 nor CB1 IAs affected CBC + THC reduction of cell motility. Hence, neither CB1 nor CB2 appear to be involved in CBC + THC activity on cell motility. 

Cell motility is dependent on F-actin integrity [17]. Examination of F-actin in cells treated with CBC + THC or CBD resulted in changes to F-actin structures, suggesting effects on F-actin organization and disintegration. However, this needs to be further examined since changes to actin structures as observed may represent nucleation sites or actin reservoirs. Disintegration of the cytoskeleton may indeed lead to reduction in cell motility as observed in the treated cells [34,35]. 

CBC + THC treatment also led to F-actin accumulation in the cell periphery. In some cases, intensive F-actin accumulation at the tips of the cell periphery increased cell invasion (determined as cell vertical movement across the membrane), such as in, for example, invading MDA-MB-231 breast cancer cells [36]. Nevertheless, CBC + THC and CBD treatments inhibited rather than increased T24 cell vertical movement to some extent in comparison to the vehicle control, suggesting that one, cell migration might be independent of “leading edge” signaling (i.e., actomyosin contractility at the back of the migrating cell). An increase in filamentous actin at the rear of the cell does not necessarily correspond to the generation of lamellipodia on the leading edge, as required for cell migration [37]. In agreement with the above, our results suggest that the F-actin accumulation on the cell periphery in CBC and CBC + THC treatments is not associated with enhanced cell movement. 

Two, horizontal cell migration may rely on a mechanism that varies from that of vertical cell movement; both associated with cell invasion. For example, vascular epithelial cells deficient in small GTPase cell division cycle 42 (Cdc42) activity may segregate horizontally but not vertically [38]. Interestingly, CBC + THC treatment inhibits horizontal migration more than MMC, but MMC treatment has a greater inhibition of vertical cell movement. This suggests that each treatment has a greater effect on one cell-movement mode than the other does.

To conclude, APIs from the cannabis extract of a high-CBD strain were found to have cytotoxic and synergistic activity in vitro against UC cell lines, one which involves cell cycle arrest and apoptosis. The API treatments also reduced cell migration and affected the cell cytoskeleton. Inhibition of apoptosis and cell migration by the API may have therapeutic significance. Induction of apoptosis may reduce the number of cancer cells; reduction of cell migration may suggest an ability to reduce cancer cell invasion or the onset of metastasis. However, pre-clinical studies and clinical trials are needed to validate the efficacy of the identified API for the treatment of UC.

## 4. Materials and Methods 

### 4.1. Plant Growth and Extract Preparation

A high-CBD strain of *C. sativa* from the Israel Gene Bank (IGB) collection, was cultivated from cuttings. During the vegetative phase, plants were grown for 10 weeks under long day conditions (18 h light/6 h dark). During the generative phase, the plants were exposed to short day conditions (12 h light/12 h dark) for 7 weeks. Inflorescences were harvested when trichomes were mostly white. Ethanol extraction was carried out as described previously [39] and decarboxylation by heating the dry extract to 220 °C for 10 min. The dried extract was weighed, resuspended in absolute methanol (volume of solvent added according to the final desired concentration) and filtered through a 0.45 μm syringe filter. 

### 4.2. Extract Fractionation

Flash chromatography was carried out using a Buchi Pure C-810 Flash apparatus equipped with a diode array detector (Buchi, Flawil, Switzerland) to separate the various cannabinoids from crude cannabis extracts. 

### 4.3. Standard/Material Preparation

Restek phytocannabinoid standards were used in this study: ∆-9 tetrahydrocannabinol (Δ-9 THC, 34067), cannabichromene (CBC, 34092), cannabidiol (CBD, 34011) at a concentration of 1 mg/mL, originally dissolved in methanol. For quantification of phytocannabinoids the standards were dissolved in methanol at various concentrations from 0–25 μg/mL. Mitomycin-C (MMC) was dissolved in water at a stock concentration of 800 μg/mL. Inverse agonists (IA) CB1 (AM251, Abcam ab120088), CB2 (SR144528, Abcam ab146185), TRPA1 blocker (HC-030031, Abcam ab120554) and TRPV1 and TRPV2 antagonists (Abcam ab141772 and Tranilast 1098/10, respectively; Tranilast is a TRPV2 inhibitor [40]) were dissolved in dimethyl sulfoxide (DMSO) at a concentration of 10mM.

### 4.4. Chemical Analysis

High-performance liquid chromatography (HPLC 1260 Infinity II, Agilent, Santa Clara, CA, USA) analysis was carried out as described in [39] using isocratic separation with acetonitrile (20%) and water with 5mM ammonium formate and 0.1% formic acid (80%) at a constant flow rate of 1.5 mL/min. Gas chromatograph with mass selective detector (GC/MS 8860 GC/5977BMSD, Agilent, Santa Clara, CA) analysis was carried out also as described in [39]. 

### 4.5. Cell Cultures

UC cell line T24 (ATCC, HTB-4) was cultured in McCoy’s 5A (BI-01-075-1A) growth media, while UC cell line HTB-9 (ATCC, 5637) was cultured in RPMI 1640 (BI-01-100-1A). Both contained 10% fetal bovine serum (FBA, BI-04-127-1A) and 1% pen-strep (BI-03-031-1B).

### 4.6. Cell Proliferation Assay

The XTT assay was carried out as described in [39], on T24 and HTB-9 cells. 1 × 10^4^ cells/well were seeded in 96-well plates. Methanol was used as the vehicle control at the same concentration as the treatments, i.e., at concentrations that do not lead to cell death. MMC was used as positive control. Cell viability was calculated relative to the vehicle control after subtracting the blank.

### 4.7. Analysis of Combined Drug Effects

Drug synergy was determined by the Bliss independence drug interaction model as described in [39], on T24 cells.

### 4.8. Apoptosis Assay and Cell Cycle Analysis

T24 cells were treated with cannabis compounds or with methanol as vehicle control. Cell cycle was determined at 24 h and apoptosis was determined at 48 h post-treatment. Staining and detection followed manufacturer instructions [39]. Briefly, for the apoptosis assay a MEBCYTO Apoptosis Kit with Annexin V-FITC and propidium iodide (PI) (MBL, Enco, 4700) was used. Cells were seeded in 6-well plate culture dishes, at density of 5 × 10^5^ cells per well. The following day, the media was replaced with new media containing treatments and vehicle control. Following, cells were harvested and stained using 10 µL of Annexin V- FITC solution and 5 µL of PI working solution, flow cytometry performed using a FORTESA flow cytometer (FACS). Cells were considered to be apoptotic if they were Annexin V + /PI- (early apoptotic) or Annexin V + /PI + (late apoptotic). Live cells were defined as Annexin V-/PI-, and Annexin V-/PI + as necrotic. 

For determination of the cell cycle phases, cells were seeded in 6-well plate culture dishes at a concentration of 5 × 10^5^ cells per well. After 24 h of incubation, the media was replaced with new media containing treatments and vehicle control. Cells from each well were then harvested and fixed with 70% cold ethanol at 4 °C for at least 1 h. The fixed cells then were pelleted out and washed twice with 1 mL of Phosphate Buffered Saline (PBS). The cell pellet was then stained by resuspending in 250 µL of PI solution (50 µg/mL) containing RNase A (100 µg/mL) for 30 min in darkness. Then 200 µL of PBS was added to each tube and the cells were analyzed using FACS.

### 4.9. Cell Migration and Cell Invasion Assays 

For the cell migration assay T24 cells were seeded into a 96-well tissue culture plate, 2 × 10^4^ cells/well. After 24 h the cell monolayer was scratched perpendicularly across the center of the well with a 200 µL pipette tip. Immediately after scratching the culture medium was aspirated and 100 µL of treatment solution was added. Photos were taken at 0, 10, 12, 14 and 16 h following scratching, and the gap area was measured using ImageJ [41] (*n* = 12). The scratch area, indicated by cells migrated into the scratch at time *x*, was calculated as percent of scratch area at time x from time 0: $\frac{\left(x\;h\;cell\;free\;area\right)\times \;100\;}{\left(0\;h\;cell\;free\;area\right)}$.

The transwell assay was used to determine cell invasion (*n* = 3), as described in [42].

### 4.10. Cytoskeleton Staining 

T24 cell were seeded on glass cell culture dishes and fixed with 3.7% formaldehyde solution and permeabilized with 0.1% Triton X-100 at room temperature. Fixed cells were blocked in Phosphate Buffered Saline (PBS) containing 1% bovine serum albumin. For actin and nuclear staining cells were incubated with EasyProbes ActinRed 555 Stain, and Hoechst, respectively. Image acquisition was done from at least 10 optical sections using a Leica SP8 laser scanning microscope (Wetzlar, Germany), equipped with a 405 and 552 nm solid state lasers, HCX PL APO CS 10 × /0.40 or HC PL APO CS 60 × /1.2 water immersion objectives (Leica, Wetzlar, Germany) and Leica Application Suite X software (Wetzlar, Germany). Hoechst and ActinRed 555 emission signals were detected with PMT and HyD (hybrid) detectors in ranges of 415–490 and 565–660 nm, respectively. At least three images were captured from each slide, and experiments were repeated four times.

### 4.11. Quantitative Real-Time (qRT) PCR 

qRT PCR was carried out as described in [39]. Briefly, T24 cells were treated with cannabis compounds or methanol as a vehicle control for 6 h. Cells were then harvested and total RNA was isolated. RNA was reverse-transcribed. The expression of each target gene was normalized to the expression of *Hypoxanthine Phosphoribosyltransferase 1* (*HPRT1*; geneID 3251) mRNA as the 2^-ΔΔCt^ method presenting the differences (∆) in threshold cycle (Ct) between the target gene and *HPRT1* gene. ΔCt = Ct Target gene - Ct *HPRT1*. Experiments were repeated three times. The primers were: for CB1 (*CNR1*; geneID 1268) (forward) 5’- AAGACCCTGGTCCTGATCCT-3’ and (reverse) 5’- TGTCGCAGGTCCTTACTCCT-3’; for CB2 (*CNR2*; geneID 1269) (forward) 5’-ATCATGTGGGTCCTCTCAG-3’ and (reverse) 5’-GATTCCGGAAAAGAGGAAGG-3’.

### 4.12. Statistical Analysis

Means of replicates were subjected to statistical analysis by Tukey–Kramer test or Student’s *t*-test using the JMP statistical package [43] and considered significant when *p* ≤ 0.05.

## Figures and Tables

**Table 1:** The effect of the cannabinoid compounds on cell invasion.

Treatment	Cell Invasion (%) Relative to Vehicle Control
MMC 4 µg/mL	43.7 ± 4.8
CBC+THC (8.6 + 1.4 µg/mL)	90.5 ± 4.1
CBD (10 µg/mL)	89.7 ± 6.2

## Data Availability

The data presented in this study are available in supplementary material.

## Supplementary Materials

The following are available online, Figure S1: Cell viability of HTB-9 cells treated with the C. sativa IGB strain extract and respective fractions. Cell viability was determined by XTT assay as a function of the number of live cells, Figure S2: Cell viability of T24 cells treated with CBD (13.8 μg/mL) with or without CB1 (AM251) or CB2 (SR144528) inverse agonists (IA; 10 µM), Figure S3: (a) An example of FACS output following PI staining used for determining the stages of cell cycle arrest and (b) Annexin V-FITC and PI staining for determining the proportion of viable (Q4), apoptotic (Q2 and Q3 for late and early apoptosis, respectively) or necrotic cells (Q1), Figure S4: Determination of the proportions of viable, apoptotic or necrotic cells following treatment with CBC+THC (17.2 + 2.8μg/mL), respectively on T24 cell line for 24 h, Table S1: Phytocannabinoid composition of the crude extract and fractions of Israel Gene Bank strain based on HPLC analysis, Table S2: The experimental (observed) and the calculated (expected) values of the synergistic interactions between CBC and THC on cell viability of T24 cells following combined treatments.

## Sample Availability

Samples of the compounds are available from the authors subjected to authorization from the Israeli Ministry of Health and Volcani Institute committee.
