# Supplementary figures and images for: Cannabis-Derived Compounds Cannabichromene and Δ9-Tetrahydrocannabinol Interact and Exhibit Cytotoxic Activity against Urothelial Cell Carcinoma Correlated with Inhibition of Cell Migration and Cytoskeleton Organization
Source: Molecules. 2021 Jan 17;26(2):465. doi: 10.3390/molecules26020465 (PMC7830447; doi:10.3390/molecules26020465)

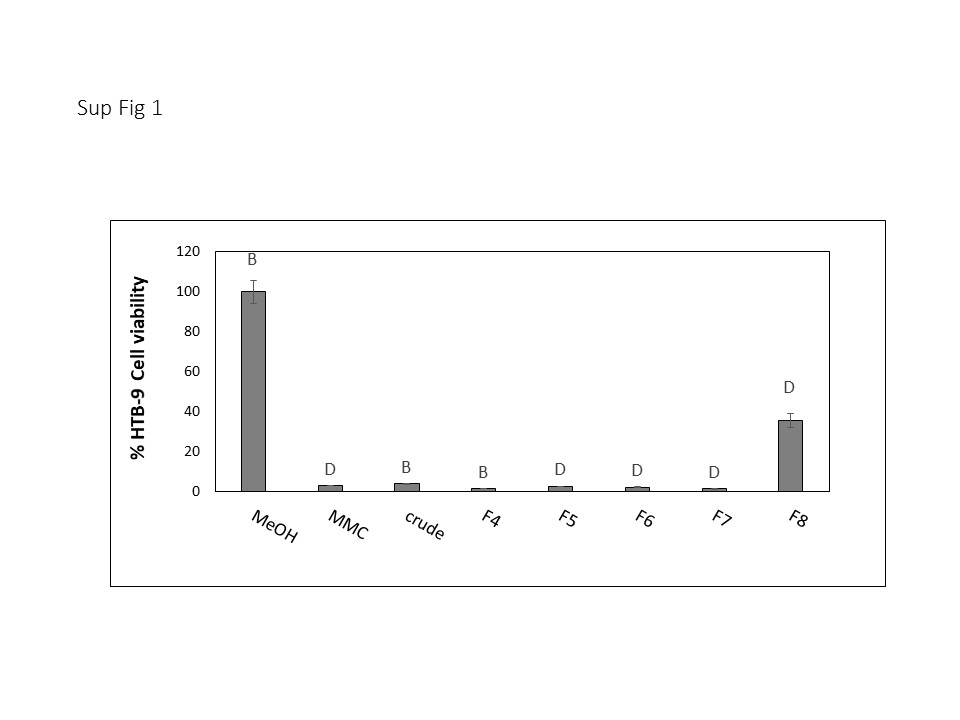

Supplement: Supplementary file 1 [file molecules-26-00465-s001.zip › sup Fig 1.jpg]

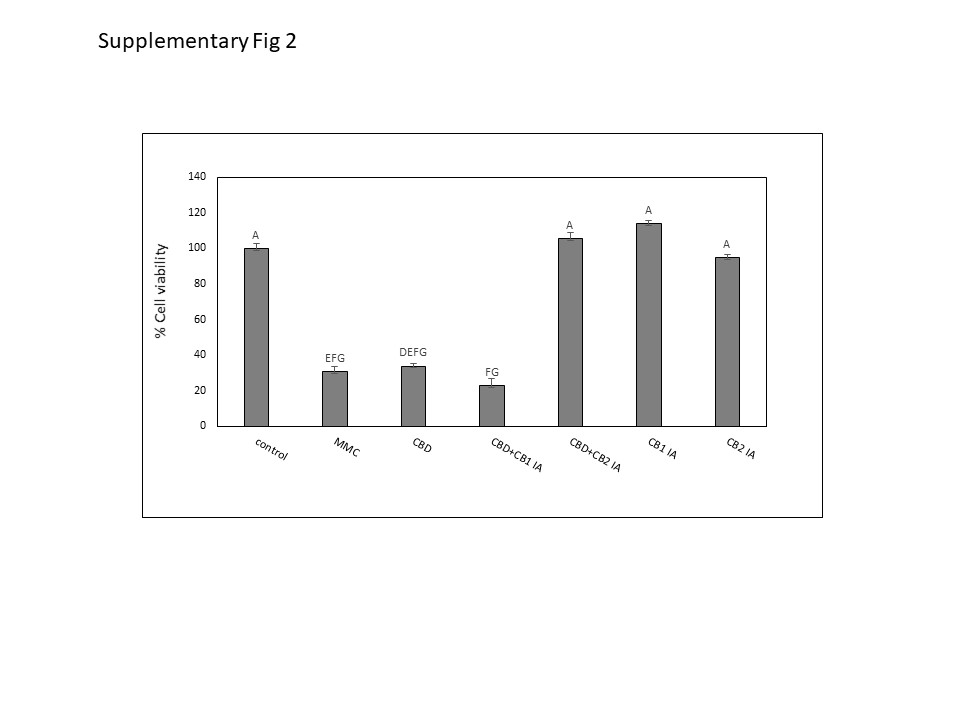

Supplement: Supplementary file 1 [file molecules-26-00465-s001.zip › sup Fig 2.jpg]

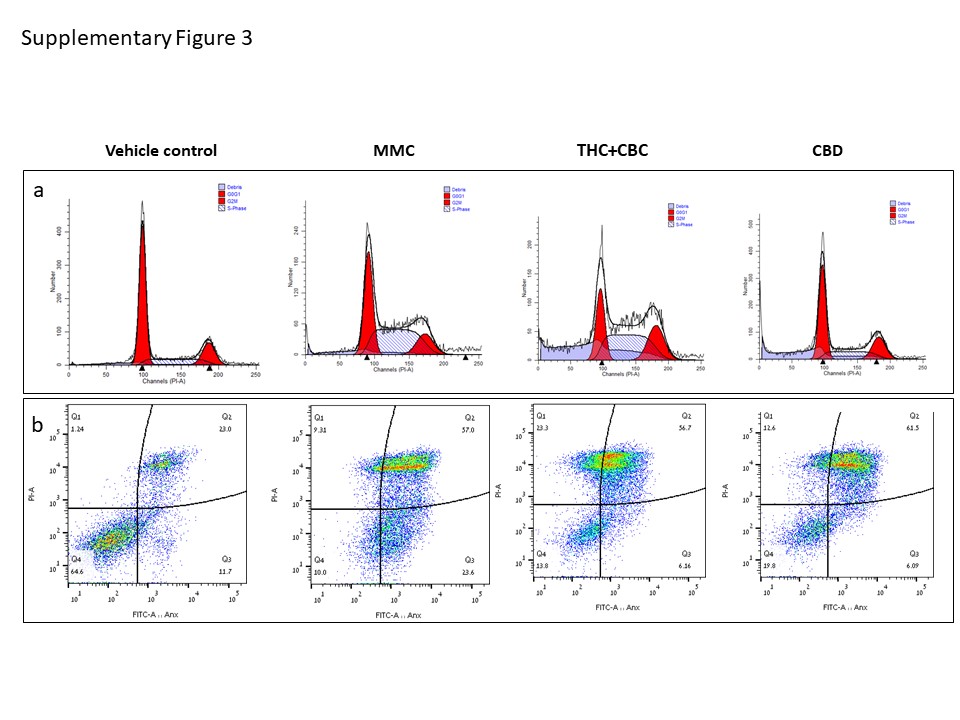

Supplement: Supplementary file 1 [file molecules-26-00465-s001.zip › Sup Fig 3.jpg]

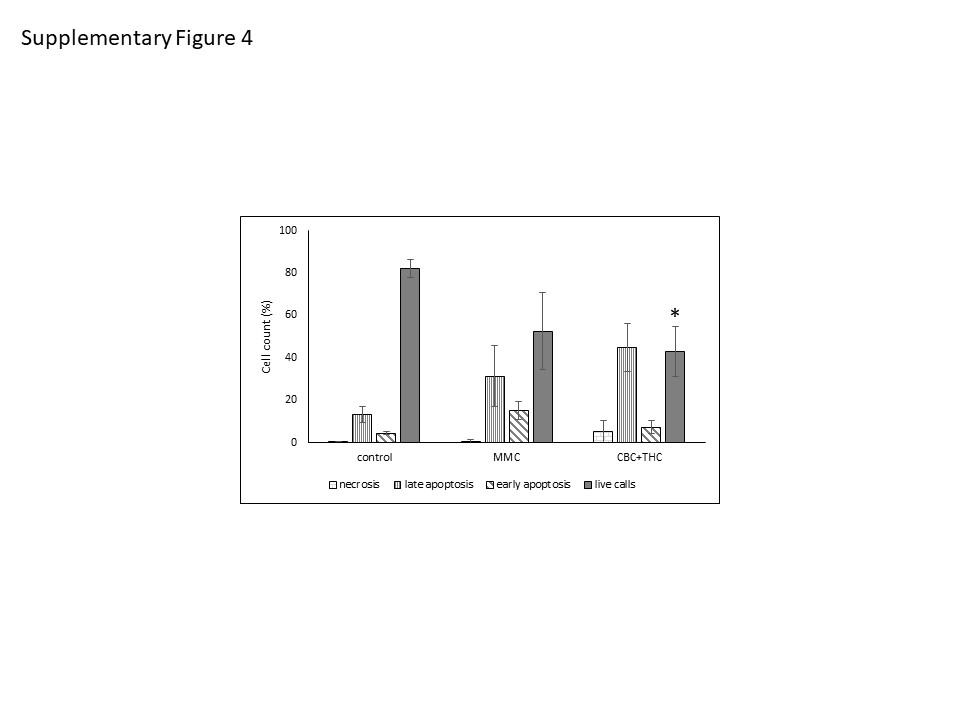

Supplement: Supplementary file 1 [file molecules-26-00465-s001.zip › Sup Fig 4.jpg]
